# Radiological assessment of anal cancer: an overview and update

**DOI:** 10.1186/s13027-016-0100-y

**Published:** 2016-10-12

**Authors:** Vincenza Granata, Roberta Fusco, Alfonso Reginelli, Luca Roberto, Francesco Granata, Daniela Rega, Antonio Rotondo, Roberto Grassi, Francesco Izzo, Antonella Petrillo

**Affiliations:** 1 Division of Radiology, Department of Diagnostic Imaging, Radiant and Metabolic Therapy, “Istituto Nazionale Tumori Fondazione Giovanni Pascale – IRCCS”, Naples, Italy; 2 Department of Internal and Experimental Medicine, Magrassi-Lanzara, Institute of Radiology, Second University of Naples, Naples, Italy; 3 Department of Civil and Mechanical Engineering, University of Cassino and Southern Lazio, Cassino, Italy; 4 Department of Colorectal Surgical Oncology, “Istituto Nazionale Tumori Fondazione Giovanni Pascale – IRCCS”, Naples, Italy; 5 Department of Surgical Oncology, “Istituto Nazionale Tumori IRCCS Fondazione Pascale – IRCCS di Napoli”, Naples, Italy

**Keywords:** Anal Cancer, 3D Endo anal Ultrasound, Magnetic Resonance Imaging, Detection Cancer, Post-treatment Imaging Assessment

## Abstract

Anal cancer is uncommon neoplasm with an incidence of 2 new cases per 100,000 per year in the USA, accounting approximately 0.4 % of all tumors and 2.5 % of gastrointestinal malignancies. An early detection of the anal cancer is crucial for the patient management, whereas the diagnosis at an early stage allows conservative management with sphincter sparing, on the contrary a delays in diagnosis might lead to an advance cancer stage at presentation with worst survival. According to National Comprehensive Cancer Network (NCCN) Anal Carcinoma guidelines the patients should be subjected to a careful clinical examination, including a digital rectal examination (DRE), an anoscopic examination, and palpation of inguinal nodes. The guidelines recommended for the assessment of T stage, only a clinical examination, while the role of imaging techniques, as Magnetic Resonance imaging (MRI) is limited to the identification of regional nodes. Instead, the endoanal ultrasound (EAUS) is not recommended. This paper presents an overview and some updates about 3D EAUS and MRI in detection, staging and assessment post therapy of anal cancer patients.

## Background

Anal caner is uncommon neoplasm with an incidence of 2 new cases per 100,000 per year in the USA [1], accounting approximately 0.4 % of all tumors and 2.5 % of gastrointestinal malignancies [2, 3]. Compared to 30 years ago, the incidence increased by about 2 fold higher (1.9 fold for men and 1.5 for women) [3]. Several risk factors have been identified: the number of sexual partners, cigarette smoking, genital warts, a history of vulvar, vaginal or cervical cancer, immunosuppression after solid transplantation, hematologic malignancies and viral infections by human papillomavirus (HPV), and human immunodeficiency virus (HIV) [3–6]. Some studies showed that the increasing incidence of anal carcinoma might reflect an increase in infection rates of HPV and HIV [5–10].

**Fig. 1:**
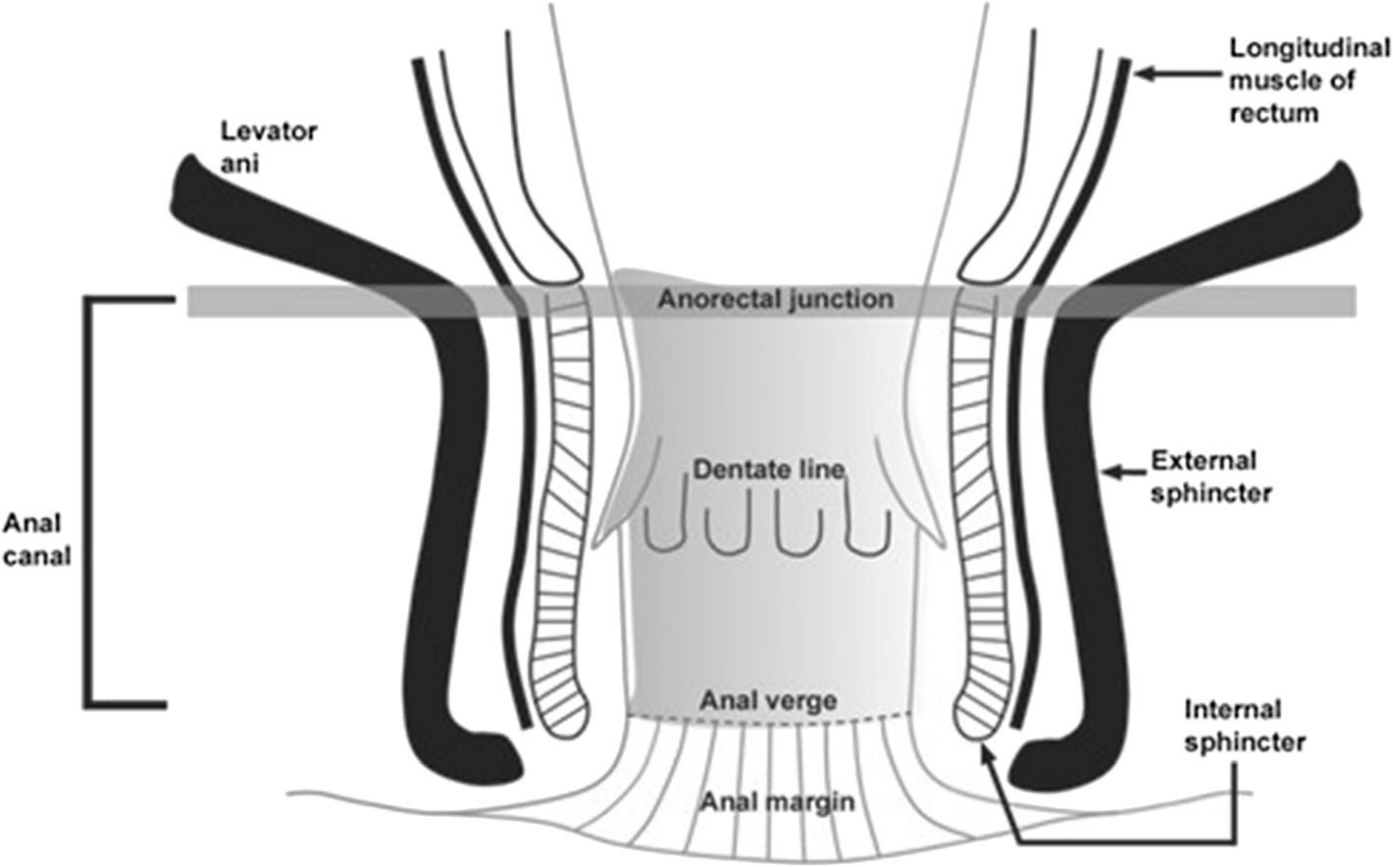
Anatomical scheme of anal canal: levator ani, longitudinal muscle o rectum, anorectal junction, dentate line, anal verge, anal margin, internal sphincter and external sphincter

Most primary cancers of the anus are squamous cell carcinomas [11]. Lymphatic drainage of tumor is dependent on location of the lesion in the anal region [11]: cancer in the perianal skin and in distal anal canal drains to superficial nodes, while tumors in the proximal anal canal drain in anorectal, perirectal, paravertebral nodes and also in internal iliac nodes (Fig. 1) [11]. Diagnosis based only on patient’s clinical history is complicated since anal tumor is characterized by a considerable overlap of symptoms with benign diseases: 45 % of patients report rectal bleeding, 20–35 % anorectal pain and 20–35 % sensation of a rectal mass [7, 8]. An early detection of the anal cancer is crucial for the patient management, whereas the diagnosis at an early stage allows conservative management with sphincter sparing. [9, 12, 13] On the contrary, a delays in diagnosis might lead to an advance cancer stage at presentation with worst survival, [8]. The recent improvements of neoadjuvant therapies, radiotherapy and chemotherapy, can also down staging the lesion, as well as to allow a conservative treatment [9–14]. According to National Comprehensive Cancer Network (NCCN) Anal Carcinoma Guidelines the patients should be subjected to a careful clinical examination, including a digital rectal examination (DRE), an anoscopic examination, and palpation of inguinal nodes [15]. The NCCN recommended for the assessment of T stage (Table 1) only a clinical examination, while the role of imaging techniques, as Magnetic Resonance imaging (MRI) is limited to the identification of regional nodes. The endoanal ultrasound (EAUS) is not recommended [15]. However, the clinical examination alone does not allow to evaluate the relationship of the tumor with structures such as sphincter plan, vagina, cervix, urethra, which is obligatory to confirm the stage of the cancer and to choose therapeutic strategy, since tumors in stage T2 and T3/T4 (Table 2) are usually treated surgically or with neoadjuvant therapy, respectively [13]. Also, NCCN guidelines recommend that the surveillance post primary treatment of non metastatic cancer should be by DRE between 8–12 weeks after neoadjuvant therapy and these patients should undergo evaluation every 3 – 6 months for 5 years by DRE, anoscopic evaluation and inguinal node palpation. For patients with slow disease regression is recommended an annual chest, abdominal and pelvic imaging [15].

**Table 1:** TNM Classification for anal cancer

Primary tumor (T)	
TX	Primary tumor cannot be assessed
T0	No evidence of primary tumor
Tis	Carcinoma in situ (Bowen disease, high-grade squamous intraepithelial lesion [HSIL], anal intraepithelial neoplasia II-III (AIN II-III)
T1	Tumor 2 cm or less in greatest dimension
T2	Tumor more than 2 cm but not more than 5 cm in greatest dimension
T3	Tumor more than 5 cm in greatest dimension
T4	Tumor of any size invades adjacent organ(s) (eg, vagina, urethra, bladder);direct invasion of the rectal wall, perirectal skin, subcutaneous tissue, or the sphincter muscle(s) is not classified as T4
Regional lymph nodes (N)	
NX	Regional lymph nodes cannot be assessed
N0	No regional lymph node metastasis
N1	Metastasis in perirectal lymph node(s)
N2	Metastasis in unilateral internal iliac and/or inguinal lymph node(s)
N3	Metastasis in perirectal and inguinal lymph nodes and/or bilateral internal iliac and/or inguinal lymph nodes
Distant metastasis (M)	
M0	No distant metastasis
M1	Distant metastasis

**Table 2:** Anatomic stage

Stage	T	N	M
0	Tis	N0	M0
I	T1	N0	M0
II	T2	N0	M0
	T3	N0	M0
IIIA	T1	N1	M0
	T2	N1	M0
	T3	N1	M0
	T4	N0	M0
IIIB	T4	N1	M0
	Any T	N2	M0
	Any T	N3	M0
IV	Any T	Any N	M1

Both the 3-dimensional (3D) EAUS and MRI allows to perform a detailed evaluation of the multilayer wall of the anus, to detect the lesion, to stage the lesion, to identify the relations with adjacent structures and the presence of lymphadenopathy [16].

This paper reports an overview and some updates about 3D EAUS and MRI in detection, staging and assessment post therapy in anal cancer patients.

## Materials and methods

Data for this review were identified by searches of the PubMed database using a multimodal strategy. The following search terms were employed: endo sonography in anal cancer, three Dimensional-Endoanal-sonography in anal cancer, Magnetic Resonance in anal cancer, functional Magnetic Resonance in anal cancer, assessment post treatment of anal cancer, Magnetic Resonance after chemotherapy in anal cancer, endo sonography after neoadjuvant therapy in anal cancer. The inclusion criteria were: clinical study evaluating anal cancer, clinical study evaluating new functional imaging criteria in the MR study of patients with anal cancer, and clinical study evaluating follow-up after chemoradiotherapy of patients with anal cancer. Articles published in the English language from January 1989 to June 2016 were included. The references of these articles were also analyzed to identify original studies that were not identified by the search of the data. Exclusion criteria unavailability of full text and absence of original research data (editorials, case reports, etc.).

## Results

A Pubmed search yielded 9 articles for key endo sonography in anal cancer, 2 articles for key 3 Dimensional-Endoanal-sonography in anal cancer, 442 articles for key Magnetic Resonance in anal cancer, 25 articles for key functional Magnetic Resonance in anal cancer, 31 articles for key assessment post treatment of anal cancer, 60 articles for key Magnetic Resonance after chemotherapy in anal cancer, 1 articles for key endo sonography after neoadjuvant therapy in anal cancer. According to inclusion and exclusion criteria, 26 articles were included at the end.

## Discussion

### Dimensional-Endoanal-sonography

#### Detection and staging

**Fig. 2:**
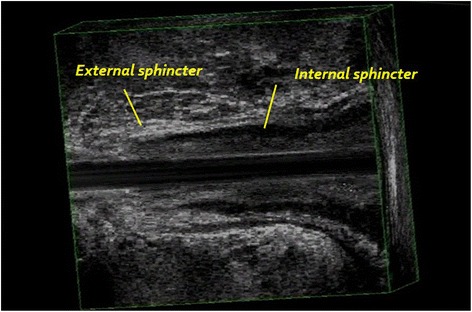
3D EAUS: longitudinal plane; external and internal sphincter

**Fig. 3:**
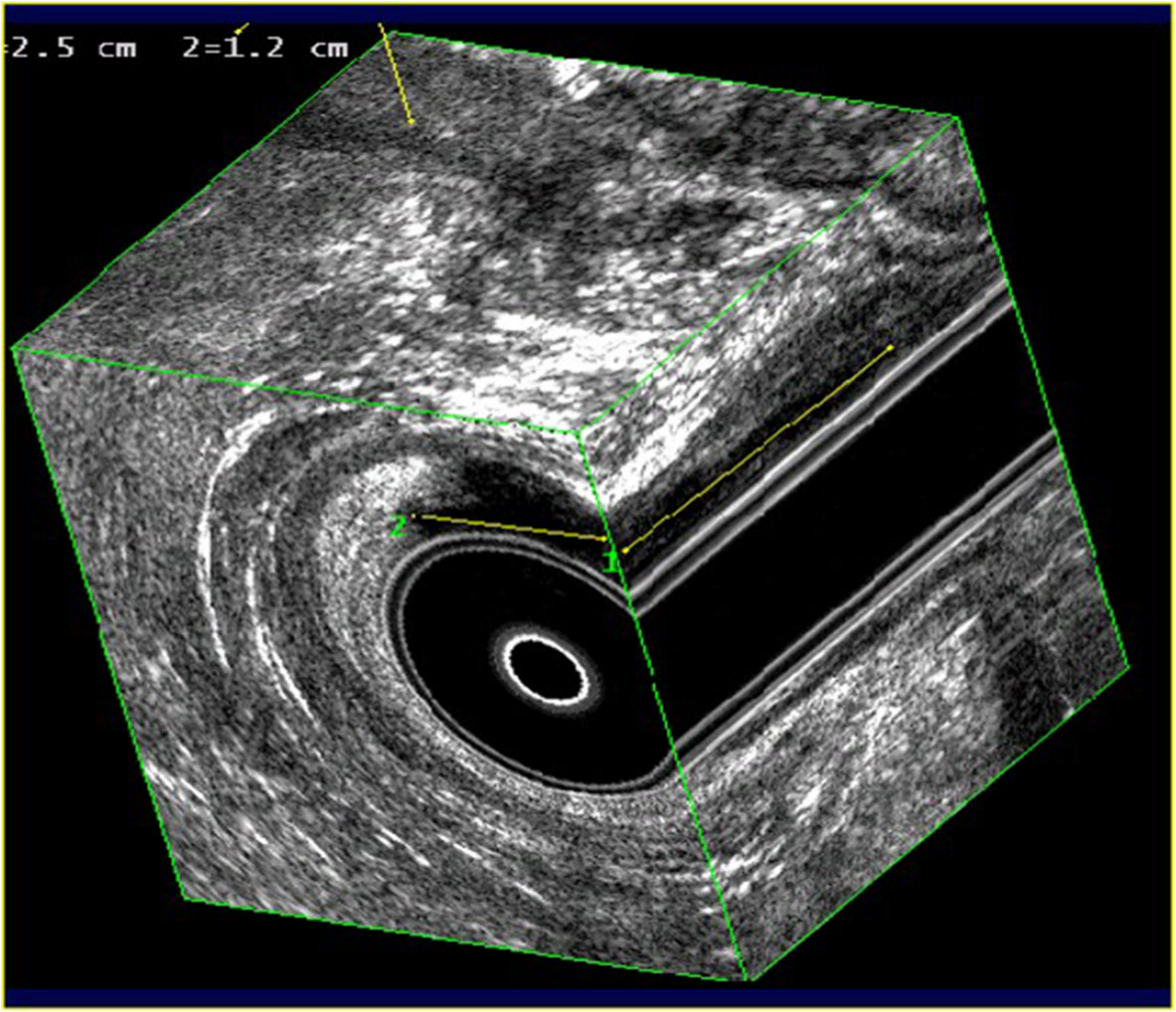
3D EAUS: tridimensional reconstruction

3D-EAUS is a valuable tool to evaluate the normal anatomy and diseases of the anal canal (Fig. 2). It is easy to perform and to reproduce, with high diagnostic accuracy. It is painless, without patient preparation. It provides excellent imaging of the rectal and anal wall, of the internal and external sphincters (Fig. 3), of the intersphincteric plane, and of the position of the anal verge, essential for planning surgical approach. 3D-EAUS is the first investigation in benign anal diseases [17]. As showed by Alabiso et al. [17] it is a diagnostic method able to identify the intersphincteric or submucosal lesion, depicting the intersphincteric plane and both the internal and external sphincters, whose identification is critical to the proper patients management [12]: to identify a T1 lesion allows conservative management with sphincter sparing. Kolev et al. [18] demonstrated that T category on 3-D EAUS correlated with histopathology in 92.9 %, and N category correlated with histopathology in 81.6 %, so the 3D EAUS is a valuable diagnostic tool in the assessment of the anal cancer, even for stage T1 [18]. Christensen et al. in 2004 compared 3-D EAUS with 2-D EAUS and showed that 3D improved detection of perirectal nodes, becoming a powerful tool in staging and planning of treatment [19]. Also Kim [20] analyzed the role of EAUS showing that it can accurately detect the depth of anal cancer into the sphincters with a focus on tumor penetration. This aspect is important because the depth of penetration is closely associated with the prognosis, demonstrating that EAUS may be superior for the detection of superficial small anal cancers compared to MRI and therefore recommended for T staging. For lymph node staging, Kim showed that EAUS should be supplemented by MRI, since US has a limited field of view [20]. In addition, Regadas et al. [21] showed that 3D EAUS, with high spatial resolution, could be a valuable tool to diagnose anorectal diseases, adding important features about the therapeutic decision. Although the diagnostic performance is similar to that of MRI, 3D EAUS has the advantages of being easier, quicker, cheaper and better tolerated by patients than the other. The 3D EAUS has a role not only in detection and staging, but also as a guide to treatment. In fact, Christensen et al. [22] showed that 3D EAUS could lead to brachytherapy in anal carcinoma, demonstrating how it could optimize the implant procedure and offer better information for dose planning.

#### Post treatment assessment

The advent of new therapies, more and more personalized to the patients and the recent improvements of neoadjuvant therapies, radiotherapy and chemotherapy can cause a down staging of the lesion, such as to make operable lesions that were not resectable before treatment [12–14]. In addition, the choice of low-invasive surgery, with sphinter saving technique, increases the risk of recurrence. Therefore is mandatory identifying effectiveness of neoadjuvant therapy and of the recurrent disease. In this context, the role of 3D EAUS is unclear. Christensen et al. [23] showed that the 3D EAUS was an accurate technique in detection of recurrence of anal cancer in combination with anoscopic and digital rectal examination post surgery. Conversely, Peterson et al. [24] demonstrated that EAUS did not provide any advantage compared to DRE in identifying local recurrence, and should not be recommended for routine surveillance. To the best of our knowledge, there are no scientific evidences of effectiveness of 3D EAUS in the assessment of anal tumor after neoadjuvant therapy.

3D-EAUS has some limitations since it is highly operator dependent and it does not allow a reliable distinction between tumor and fibrosis.

### Magnetic resonance imaging

#### Detection and staging

**Fig. 4:**
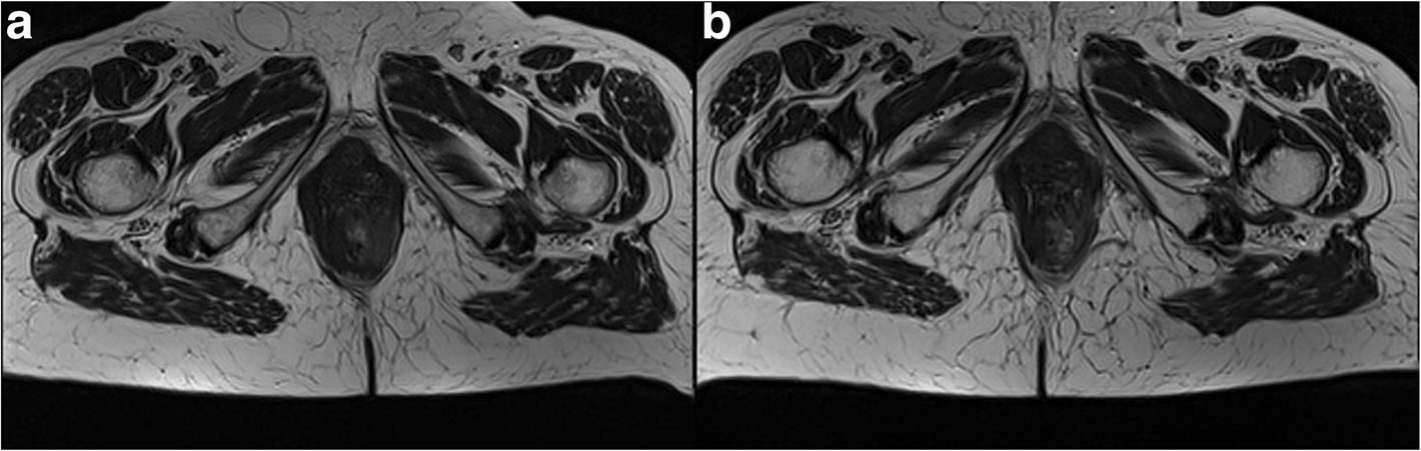
TSE T2-W in axial plane; (**a**) anal cancer infiltrating internal and external sphincter on the left; inguinal node. **b** post treatment assessment: partial response with involvement of internal sphincter; inguinal node disappearances

**Fig. 5:**
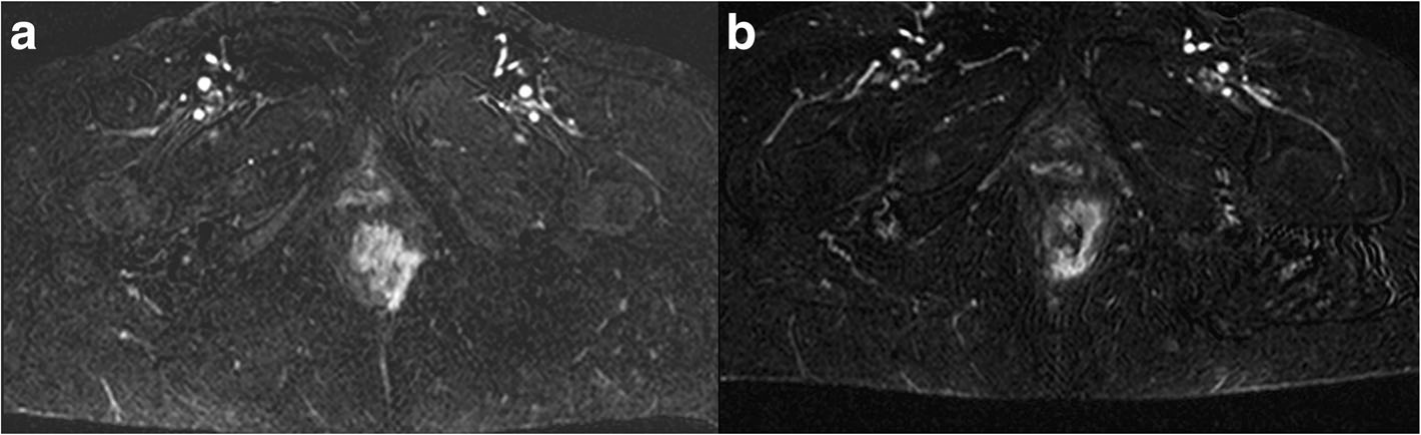
Same patient of 4: post contrast sequences; in (**a**) pre treatment: contrast enhancement of anal cancer infiltrating internal and external sphincter on the left. In (**b**) post treatment assessment: the lesion shows a lower contrast enhancement

**Fig. 6:**
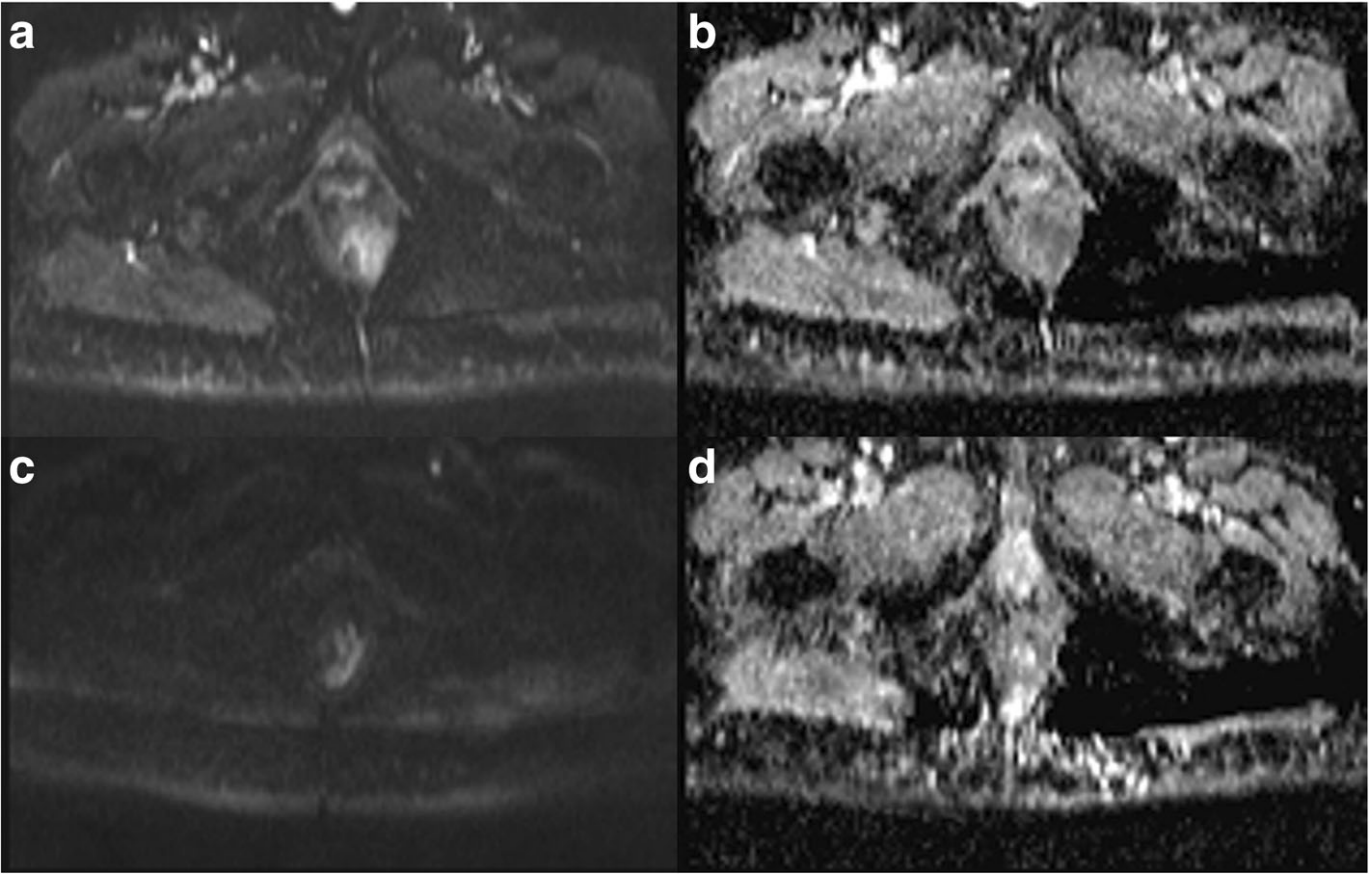
Same patient of 4 and 5: DWI sequences. In (**a**) b800 pre-treatment examination: cancer shows hyperintese signal, in (**b**, **a**, **d**, **c**): the lesion appear hypointhense. In (**c**) post treatment b800, a lower signal than in A with a higher signal in (**a**, **d**, **c**) than in **b**

MRI is the gold standard in oncological pelvic examination, providing morphological and functional data. MRI leads to an excellent imaging of the rectal and anal wall, so to obtain an accurate evaluation of cancer stage including tumour infiltration degree, involvement of the internal and external sphincters, of the intersphincteric plane, and an effective assessment of lymph nodes status thanks to improvement of phased array coils and endorectal coils. A standard MRI protocol for anal cancer staging consists of turbo spin-echo MR sequences T2-weighted in the 3 spatial planes (coronal, transversal and sagittal) with high spatial resolution (Fig. 4) [25, 26]. Moreover, the high temporal resolution due to powerful gradients allows perfusion and dynamic studies after contrast media injection, in order to obtain functional data to assess the type of tissue blood supply, which may guide patient selection for neoadjuvant therapies and evaluate the treatment [25, 26]. So that a dynamic study acquired according to the technique Dynamic Contrast Enhanced (DCE) should be performed (Fig. 5). Moreover, functional parameters can be obtained by Diffusion Weighted Imaging (DWI). DWI supplies information of water mobility. This can be employed to assess the microstructural organization of a tissue like cell density, cell membrane integrity and ultimately cell viability [27]. An anal cancer MRI protocol should be also performed with DWI sequence (Fig. 6).

MRI is a valuable diagnostic tool in anal cancer staging, although the major limitation is an incorrect detection of T1 patients [16], neither it would seem that the use of the endoanal coil could increase the detection rate [27]. In fact Matsuoka et al. [27] demostrated that endorectal coil and phased array coil showed similar diagnostic accuracy in detection of anal cancer. Several studies evaluated the MRI accuracy compared to EAUS, in rectal cancer patients staging, and the data suggested that EAUS provides an excellent visualization of the layers of the bowel wall conversely to MR so that EAUS provides better detection of superficial tumor. In evaluation of perianal and perirectal nodes, the techniques are complementary tool, while MR should be chosen for iliac and inguinal nodes [28–33]. Also, Burdan et al. [33] showed that the possibility to obtain functional information by MR as the increased signal on DWI and low apparent diffusion coefficient (ADC) values seem to predict the involvement of pelvic lymphatic nodes better than their size alone. According to Attenberger et al. [34], ADC measurements were useful in differentiating N stages. Mizukami et al. [35] reported a high negative predictive value ofDWI, while Heijnen et al. [36] showed that DWI increases the detection rate of lymph node, but alone it is not reliable for differentiating between benign and malignant nodes. Recently, Seber et al. [37] evaluated the DWI compared with morphological MRI for the differentiation of benign from malignant nodes in different regions of the body. They concluded that morphological data was better than DWI data alone or associated to morphological data, however DWI and ADC might play a role in node characterization.

Although the most widely used imaging tool for guidance of interstitial radiotherapy in anal cancer brachytherapy (BT) is EAUS [22], since it provides a good target delineation and a very easy administration, however also MRI is characterized by excellent contrast and spatial resolution, providing information on tumor size, local extent and spreading, invasion of adjacent organs, and more accurate nodal involvement. Furthermore, due to the use of perfusion and diffusion data, MRI can also provide information concerning functional characteristics and microcirculation of the tumor. These features could be useful for the modulation of the target dose and the improvement of the treatment results. Tagliaferri et al. [38] evaluated the role of MRI in anal BT and they showed that although the use of MRI had some limitations, such as costs and higher time required, it could be the preferable choice for the modulation of the target dose according to perfusion and DWI parameters. In fact, the functional features could help to deliver a very high dose only to a small volume, in order to reduce the dose, resulting in lower toxicity and increased local control (Table 3).

**Table 3:** MRI anal cancer features

Sequences	SI
T2-W	Hyperintense
DWI	Hyperintense
ADC map	Hipointense
T1-W	Hypointense
T1-W post mdc ev	Contrast Enhancement

#### Post treatment assessment

MR imaging plays an important role in therapeutic assessment, properly stratify patients into responders or non responders to neoadjuvant treatment, surveillance after surgery, and evaluation of suspected disease fall-out [25, 26]. The possibility to obtained functional data by DCE-MRI and DWI allows to relive vitality tissue and to differentiate fibrosis by residual tumour after anti-angiogenetic treatments [25, 26].

In clinical practice, the recognized criteria for the assessment after therapy are response evaluation criteria in solid tumor (RECIST), which are based on size criteria. Although the morphological evaluation on MR images can identify a patient as responder to therapy, based on decreased of maximum diameter of the lesion,, it does not establish if the remaining tissue is cancer or fibrosis, and it does not seem to be linked to patient outcome. Goh et al. [39] evaluated the MRI pre- and post- treatment. They showed that early assessment of response by MRI at 6−8 weeks, based on RECIST criteria, is unhelpful in predicting future clinical outcome.

Several studies evaluated the DCE-MRI as promising tool to monitor assessment after neoadjuvant therapy thanks to the link between tumor growth and angiogenesis [40, 41]. It is known that angiogenesis is a key factor in the growth of cancer, therefore the characterization of the angiogenic status of the lesions could allow a more personalize treatments [13].

Many clinical trials showed that angiogenesis inhibition could increase the treatment effectiveness. Imaging techniques is able to assess tumour vascularization and the capability to improve the treatment management in oncologic patients [40, 41].

DCE-MRI consists of a multiple T1-weigthed images acquired before and after contrast medium administration in specific time linked to study temporal resolution. It measures the rate of contrast movement between the intravascular and extra-cellular extravascular space. This rate reflects tissue microvasculature permeability and perfusion. In order to assess tissue perfusion by means of DCE-MRI, several approaches to analyse Time Intensity Curve (TIC) were proposed in literature. The most commonly used in the radiological practise is the visual inspection of TIC [25]. According to Petrillo et al. [26] the TIC visual inspection to assess neoadjuvant response in rectal cancer patients, has a sensitivity, a specificity and an accuracy respectively of 94 %, 76 % and 84 % in complete responders. Also quantitative and semi-quantitative analysis performed to evaluate vascular assessment tissues. Petrillo et al. [25] performed a semi-quantitative analysis and individuated a combination of 2 TIC descriptors, ∆MSD (relative change of maximum signal difference) and ∆WOS (relative change of wash-out slope), named Standardized Index of Shape (SIS) [25]. This combination reached a sensitivity of 93.5 % and a specificity of 82.1 %. Moreover, SIS improved negative predictive value to 88.5 % and positive predictive value to 89.6 %. Jones et al. [42] performed multiparametric MRI, including morphological, DWI and DCE sequences, to determine whether the early changes in multiparametric parameters, especially ADC and quantitative parameters, Ktrans and Kep, during neoadjuvant therapy can predict for later response in anal. However, according to Torkzad et al. [43] the real role of DWI and DCE MRI in post treatment assessment remains to be established, requiring a greater number of scientific studies.

## Conclusion

Although the anal caner is rare neoplasm, the incidence of the tumor shows an incremental trend that reflects an increase in infection rates of HPV and HIV and immunosuppression state. The real rule of imaging techniques in detection, staging and follow-up of tumor is unclear. 3D EAUS and MRI are the best diagnostic tools in detection of the lesion, although 3D EAUS is more accurate than MRI for T1 stage (Table 4). However, the MRI allows to properly detect neoplastic nodes both to higher field of view and functional data. The MRI is the techniques of choice in post neoadjuvant treatment to properly stratify patients into responders or non responders.

**Table 4:** Advantages and Weaknesses 3D EAUS versus MRI

Technique	3D EAUS	MRI
Advantages	Easier; quicker; low cost and better tolerated by patients	MRI is the gold standard in oncological pelvic examination, providing morphological and functional data
Disadvantages	The accuracy of US varies according to the operator skill; small field of view	Expensive, poorly tolerated by the patient, long time for the examination
T stage	More accurate for T1 than MRI and to asses relationship between lesion and sphincteric plan	MRI is a valuable diagnostic tool in anal cancer staging, although the major limitation is an incorrect detection of T1 patients
N stage	Only N1, so EAUS should be supplemented by MRI since US has a limited field of view.	Effective assessment of lymph nodes status thanks to morphological and functional data by DWI.
Post Treatment Assessment	EAUS did not provide any advantage over DRE in identifying local recurrence, and should not be recommended for routine surveillance	MR imaging plays an important role in therapeutic assessment, properly stratify patients into responders or non responders to neoadjuvant treatment, surveillance after surgery, and evaluation of suspected disease fall-out

## Abbreviations

ADC: Apparent diffusion coefficient
BT: Brachytherapy
DCE-MRI: Dynamic contrast enhanced-MRI
DRE: Digital rectal examination
DWI: Diffusion weighted imaging
EAUS: Endoanal ultrasound
HIV: Human immunodeficiency virus
HPV: Human papillomavirus
MRI: Magnetic resonance imaging
MSD: Maximum signal difference
NCCN: National comprehensive cancer network
RECIST: Response evaluation criteria in solid tumor
SIS: Standardized Index of Shape
TIC: Time intensity curve
WOS: Wash-out slope
